# Effect of Time of Day on Performance, Hormonal and Metabolic Response during a 1000-M Cycling Time Trial

**DOI:** 10.1371/journal.pone.0109954

**Published:** 2014-10-07

**Authors:** Alan Lins Fernandes, João Paulo Lopes-Silva, Rômulo Bertuzzi, Dulce Elena Casarini, Danielle Yuri Arita, David John Bishop, Adriano Eduardo Lima-Silva

**Affiliations:** 1 Sports Science Research Group, Federal University of Alagoas, Maceio, Alagoas, Brazil, and Department of Physical Education and Sports Science, CAV, Federal University of Pernambuco, Vitória de Santo Antão, Pernambuco, Brazil; 2 Endurance Performance Research Group, School of Physical Education and Sport, University of São Paulo, São Paulo, Brazil; 3 Nephrology Division, Department of Medicine, Federal University of Sao Paulo, São Paulo, Brazil; 4 Institute of Sport, Exercise and Active Living, Victoria University, Melbourne, Australia; Qingdao Agricultural University, China

## Abstract

The aim of this study was to determine the effect of time of day on performance, pacing, and hormonal and metabolic responses during a 1000-m cycling time-trial. Nine male, recreational cyclists visited the laboratory four times. During the 1^st^ visit the participants performed an incremental test and during the 2^nd^ visit they performed a 1000-m cycling familiarization trial. On the 3^rd^ and 4^th^ visits, the participants performed a 1000-m TT at either 8 am or 6 pm, in randomized, repeated-measures, crossover design. The time to complete the time trial was lower in the evening than in the morning (88.2±8.7 versus 94.7±10.9 s, respectively, p<0.05), but there was no significant different in pacing. However, oxygen uptake and aerobic mechanical power output at 600 and 1000 m tended to be higher in the evening (p<0.07 and 0.09, respectively). There was also a main effect of time of day for insulin, cortisol, and total and free testosterone concentration, which were all higher in the morning (+60%, +26%, +31% and +22%, respectively, p<0.05). The growth hormone, was twofold higher in the evening (p<0.05). The plasma glucose was ∼11% lower in the morning (p<0.05). Glucagon, norepinephrine, epinephrine and lactate were similar for the morning and evening trials (p>0.05), but the norepinephrine response to the exercise was increased in the morning (+46%, p<0.05), and it was accompanied by a 5-fold increase in the response of glucose. Muscle recruitment, as measured by electromyography, was similar between morning and evening trials (p>0.05). Our findings suggest that performance was improved in the evening, and it was accompanied by an improved hormonal and metabolic *milieu*.

## Introduction

Performance during a short-distance time trial (TT), such as a 1000-m cycling TT, may be optimized when an “all-out” pacing is used [1], [2]. Recently, Hettinga et al. [3] have demonstrated that the difference between the fastest and the slowest performance times for four 1500-m cycling TT (a few seconds longer than a 1000-m cycling TT) was related to a higher aerobic peak power, and a higher and earlier anaerobic peak power during the fastest trial. These authors suggested that athletes paced themselves according to their optimal physiological condition at that moment (fastest and slowest), indicating that athletes may be able to effectively adjust their pacing profile based on their “status of the day” [3]. It is interesting to observe that there is some evidence that the contribution of the aerobic and anaerobic systems is increased in the evening compared with in the morning [4], [5], suggesting that the time of day may be a potential factor influencing both energy system distribution and pacing, although there are no studies that have directly tested this relationship. The effect of time of day on pacing may be more notable during a short-distance cycling TT, in which pacing may be determined by the interplay between aerobic and anaerobic responses to the exercise.

To date, studies investigating the influence of time of day on performance have concentrated on intermittent, Wingate or constant-load exercise [4]–[9], but pacing has not been formally reported. Giacomoni et al. [7] analyzed the effect of the time of day (morning 8–10 am and evening 5–7 pm) on performance during an all-out, intermittent exercise (10 maximal 6-s sprints interspersed with 30 s of recovery) and observed a higher crank peak torque in the earlier sprints during the evening, but the overall mechanical work was not altered, suggesting that the earlier peak torque in the evening does not affect overall performance [9]. Corroborating these findings, Hill et al. [4] showed a higher total work performed during a constant-power cycle ergometer test (5.0 W·kg^−1^ for women and 6.0 W·kg^−1^ for men) in the afternoon compared with the morning, and that this greater work was associated with a larger anaerobic and aerobic contribution. The underlying mechanism explaining the greater energy expenditure and consequently improved performance in the evening is not fully understood, but a slightly increased core temperature in the evening could increase nerve conduction velocity and vasodilatation, which in turn would increase muscular supply and substrate elimination, thereby improving glycogenolysis and glycolysis [6]. While these results would support that performance during a short-distance cycling TT might be increased in the evening, due to an increase in the aerobic and/or anaerobic energy expenditure, to the best of our knowledge no study has measured the effect of time of day on performance and associated pacing alterations during a TT.

There is also limited research connecting circadian variations in hormone levels with pacing [10]. It has been demonstrated that epinephrine and norepinephrine have an ∼12-h circadian rhythm with two large peaks at 7:00–10:30 am and 8:00–10:00 pm, but with the largest exercise-induced increase in epinephrine ocurring at ∼8:30 am [11]. Cortisol and testosterone also peak at ∼8:00 am, but do not seem to respond to exercise [12]. On the other hand, growth hormone (GH) seems to be slightly reduced in the morning, but its response to exercise is not altered [13]. In addition, the effect of circadian rhythm on insulin and glucagon, two hormones that are strongly linked with metabolic *milieu* and could influence performance during different times of day, is unknown. Given that performance during a short-distance cycling TT is dependent on the ability of athletes to produce and maintain a high level of power output throughout the test [14], the coordinate action of these hormones might be associated with an improved or impaired performance. For example, epinephrine and norepinephrine prepare the body for an immediate action increasing several physiological markers (e.g. heart rate, blood pressure, metabolic rate, and glycogenolysis and glycolysis), while cortisol increases general physiological stress and, together with glucagon and GH, increases blood glucose. Elevated testosterone concentration may be involved with an increased neuromuscular efficiency and force output [15]. Thus, an improved performance at a given time of day might be associated with hormonal and metabolic at rest and/or during the exercise. However, while circadian rhythms for some hormones have been relatively well characterized [11]–[13], their integrated responses and association with TT performance have not been clarified.

The present study therefore aimed to investigate performance, pacing, energy system and muscle recruitment distributions, and hormonal responses, during a 1000-m cycling TT performed in the morning (8:00 am) or in the evening (6:00 pm). The 1000-m cycling time trial was chosen as it is energetically supported by both the aerobic and anaerobic energy systems [1], [2], and performance is influenced by pacing strategy [2], [3]. We hypothesized that performance would be improved in the evening, and that this would be associated with an altered hormonal and metabolic *milieu* at rest and/or during the exercise, and a greater aerobic and anaerobic contribution.

## Materials and Methods

### Participants

Nine recreational cyclists [Mean ± standard deviation (SD): age 31±7.3 years, height 175±7.8 cm, body mass 73.5±11.6 kg, body fat 11.6±4.7%, peak oxygen uptake (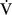 O_2_peak) 49±7 ml·kg^−1^·min^−1^, peak power output (PPO) 253±52 W] participated in this study. The participants had been training more than 3 times weekly (approximately 60 km [3–4 hours] per session) during the 4 years preceding the study, and all had good competition experience (∼14 competitions per year). The participants were classified as intermediate (n = 5) or moderate (n = 4) morning types using the chronotype questionnaire of Horne and Östberg [16]. The participants gave their written informed consent after receiving an explanation about the purpose of the study, experimental procedures and possible risks. This investigation was approved by the Ethics and Research Committee of the Federal University of Alagoas.

### Experimental design

Each participant visited the laboratory four times on different days. During the 1^st^ visit, participants completed an anthropometric assessment (body mass, height and body fat percentage calculated from skinfolds of the chest, abdomen and thigh, Jackson and Pollock [17]), and an incremental test to determine their 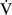 O_2_peak and PPO. At the 2^nd^ visit (after ∼48 h), the participants performed a familiarization of the 1000-m cycling TT. The 1^st^ and 2^nd^ visits were performed between 10 am and 2 pm to prevent adaptation to a particular experimental time. During the 3^rd^ and 4^th^ visits (∼96 h after the 2^nd^ visit and separated by 7 days for recovery), the participants performed a 1000-m cycling TT either in the morning or in the evening (8:00 am and 6:00 pm, respectively) in a randomized, repeated-measures, crossover design. The participants were asked to refrain from vigorous physical activity, caffeinated substances or alcohol 24 h before each test [10], [18]. The temperature and relative humidity during the trials were maintained constant and were similar between the times of day (Morning: 22.9±1.3°C and 36.3±5.9% and Evening: 23.8±0.9°C and 31.4±4.3%, respectively, p>0.05). The participants were instructed to record all foods (type, amount and time) consumed in the 48 hours before the familiarization test and to replicate this during the 48 h prior to the two experimental trials.

### Incremental test

The incremental test was performed on a cycle ergometer (Ergo Fit 167, Ergo-FitGmbH & Co., Pirmasens, Germany), and consisted of a 5-min warm-up at 100 W, followed by increments of 30 W every 3 min until voluntary exhaustion or when the participants were not able to maintain their pedal frequency between 80–90 revolutions per minute (rpm). The ventilation (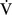 E), 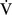 O_2_ and carbon dioxide production (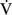 CO_2_) were measured breath-by-breath throughout the test using a gas analyzer (Quark b^2^, Cosmed, Italy). The volume of expired air was measured by a bidirectional flow sensor, calibrated before starting the test with a syringe containing 3 liters of air (Cosmed, Italy). The fraction of expired O_2_ was analyzed with a zirconium sensor and end tidal CO_2_ by infrared absorption. Both sensors were calibrated automatically before starting the test with a cylinder containing known concentrations of O_2_ (16%) and CO_2_ (5%). The HRmax and 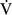 O_2_peak were taken as the highest value reached in the last stage and as the mean value obtained during the last 30 s of the test, respectively. The PPO was determined as the highest PO attained during the incremental test. When the participants could not maintain the power output during the entire stage (<3 minutes), the PPO was calculated using fractional time supported in the last stage multiplied by the increment rate (i.e. 30 W) [19].

### Familiarization trial

The familiarization session for the 1000-m TT was performed on a previously validated cycle simulator [20] and calibrated in accordance with the manufacturer's recommendations (cycle ergotrainer, Tacx T1680 Flow, Netherlands). The seat was adjusted vertically and horizontally for each cyclist before the TT, and cycling shoes were used to secure the feet to the pedals. The seat position was recorded and replicated during the subsequent experimental sessions.

### Experimental 1000-m cycling time trials

In the morning, the participants performed the 1000-m cycling TT after an 8-h overnight fast. In the evening, the participants performed the trial after a 6-h fasting period. These procedures have been recommended by Souissi et al. [5] to avoid any influence of pre-exercise diet on exercise-induced physiological response. The participants were recommended to maintain the same dietary patterns 48 hours before each test using their food records. Compliance with the directions relating to diet and water consumption was checked by food records before each experimental test.

**Figure 1:**
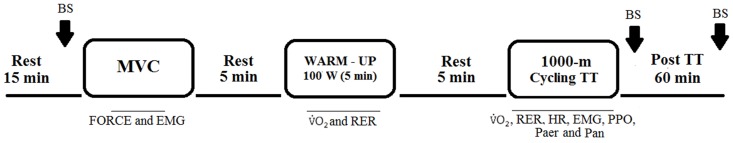
Experimental Protocol. BS: blood sample; MVC: maximal voluntary contraction; EMG: electromyographic activity; 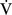 O_2_: oxygen uptake; RER: respiratory exchange ratio; HR: heart rate; PPO: power output; Paer: aerobic mechanical power output; Pan: anaerobic mechanical power output.

The experimental procedures are illustrated in Figure 1. Before each test, the participants were instructed to remain quiet for at least 15 minutes. The forearm was then cleaned with hydrated ethyl alcohol, and an intravenous catheter (IV Catheter Pen-like Model, 20 Gauge) coupled to an extender (Equipo multipath with 2-Way Clamp) was inserted. The 1^st^ blood sample (1.5 ml) was drained and discarded, and 20 ml of blood was then collected (baseline). After each blood sample collection, 1 ml of sterile sodium chloride (NaCl 0.9%) was injected to prevent clotting and the obstruction of blood through the stent.

Following the resting blood collection, in order to normalize the electromyographic activity (EMG) data during the cycling TT, three 5-s, two-legged maximum voluntary contractions (MVC) of the knee extensors (trunk-thigh angle at 90 and knee at 60° from full leg extension 0°), separated by a 60-s interval, were performed. A standardized knee extensor warm up, consisting of four 5-s contractions at intensities corresponding 50, 60, 70 and 80% of the maximum subjective force (30-s rest periods between the repetitions), was performed immediately before the MVC. The participants were verbally encouraged during the MVC to achieve their maximal force. Force was recorded using a load cell (EMG System of Brazil, São José dos Campos, Brazil). Electromyographic activity signals from the vastus lateralis (VL) of the right leg were recorded via bipolar Ag-AgCl surface electrode at an interelectrode distance of 20 mm. The VL was chosen because it has been reported as the most appropriate to monitor EMG activity in the lower limb during a cycling TT [3]. The reference electrode was placed over the anterior surface of the tibia. The skin preparation, placement and location of the electrodes were in accordance with the recommendations of SENIAM [21]. To prevent movement artifact, the electrodes' wires were taped to the skin using adhesive tape (Micropore 3 M, São Paulo, Brazil). Raw EMG signal was recorded during 5 seconds, with a sample rate of 2000 Hz, for each MVC (model 410c EMG System of Brazil Ltda, São José dos Campos, Brazil). During the MVC and TT, raw EMG signals were full-wave rectified and filtered with second-order, Butterworth, band-pass filters with cut-off frequencies set at 10 and 400 Hz to remove external interference noise and movement artifacts. Integrated EMG (iEMG) obtained every 200 m during the TT was normalized by dividing by the iEMG calculated at the point coinciding with peak torque of the highest MVC. The procedures were performed using MATLAB software.

After completion of the MVC procedures, the participants were transferred to the cycle ergotrainer. The participants remained at rest for 5 minutes before performing a 5-min warm-up at 100 W followed by a 5-min recovery (Figure 1). Thereafter, the participants were instructed to perform a 1000-m TT in the shortest possible time. The gear ratio was standardized at the beginning of each TT (53×16), but participants were free to change the gear and pedal frequency as desired immediately after the TT had started. The participants were instructed to remain seated throughout test. Feedback of the distance covered was provided every 200 m. Power output and distance were recorded at a frequency of 1 Hz (Tacx Trainer software 3.0, Wassenaar, Netherlands). The 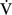 O_2_, respiratory exchange ratio (RER), HR, EMG (5-s recordings), and aerobic and anaerobic mechanical power output (Paer and Pan, respectively) were also determined and statistically analyzed every 200-m. The Pan and Paer were calculated from the RER, 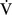 O_2_, and the efficiency estimated during the warm up, in accordance with Hettinga et al. [3].

Additional blood samples (20 mL venous blood) were collected immediately and 60 minutes after the TT (Post-TT and 60′-TT, respectively). Blood samples were transferred into tubes with EDTA (10 ml) or without non-EDTA (10 ml) and were immediately centrifuged at 3000 revolutions per minute (RPM) at 4°C for 10 minutes to separate plasma or serum, and stored at −80°C until subsequent analysis. The following hormones and metabolites were measured in the plasma: Glucagon (Radioimmunoassay, Kit Merck Millipore Standard, GER), and lactate and glucose (Spectrophotometry, kit Biotecnica, Varginha, Brazil). Plasma norepinephrine and epinephrine concentrations were measured by HPLC using the ion-pair reverse phase chromatography coupled with electrochemical detection (0.5 V), as described previously [22]. The following hormones were measured in the serum: cortisol (chemiluminescence, Kit Roche Standard, GER), growth hormone (GH) (chemiluminescence, kit Siemens Standard, GER), insulin (electrochemiluminescence, Kit Roche Standard, GER), total and free testosterone (electrochemiluminescence, Kit Siemens Standard, GER).

### Statistical analysis

The sample size required was estimated from the equation n = 8e^2^/d^2^ [23], where n, e, and d denote predicted sample size, coefficient of variation, and the magnitude of the treatment effect, respectively. Coefficient of variation to a similar time-trial was assumed to be 0.9% [24]. Detection of a very conservative 1% difference as statistically significant would require at least 6 participants. Considering possible dropouts during the study, the sample was inflated by 50%, resulting in a final sample size of 9 participants. Data distribution was analysed using the Shapiro-Wilk test. Insulin, growth hormone, cortisol, total and free testosterone, epinephrine, nor epinephrine, and glucose were transformed into natural log values as they did not satisfy the assumptions of normality. A two-way analysis of variance (ANOVA), with repeated measures (period × time), followed by Bonferroni adjustment, was used to compare the physiological, metabolic and hormonal response during the trials. When violations to the assumptions of sphericity were observed, the degrees of freedom were corrected using Greenhouse-Geisser corrections. The student test *t* was used to compare the time to complete the task between morning and afternoon and to access any possible test order effect (test 1 versus test 2). Confidence intervals (95% CI) and effect sizes for the *t* and F ratio, expressed as partial eta-squared (*η*_p_^2^), were also calculated when appropriate to evaluate the magnitude of differences. A *η*_p_^2^ of 0.1, 0.3 and 0.5 were considered as small, moderate and large effects, respectively [9]. Data are reported as means and standard deviation (M ± SD). Statistical significance was set at p≤0.05, while a trend was noted when p<0.10 (1). All analyses were performed using SPSS (17.0) software.

## Results

### Performance, energy systems contributions and physiological response to the 1000-m cycling TT

The mean values for performance time, PO, Paer, Pan, iEMG, 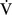 O_2_ and HR during the 1000-m cycling TT in the morning and evening are displayed in Table 1. The time to complete the TT was shorter in the evening than in the morning (*t* (8)  = 2.331, p = 0.048, *η*_p_^2^ = 0.63, 95% CI  = 0.7 to 12.8 s). The mean values for PO, Paer, Pan, iEMG, 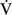 O_2_ and HR were not different between evening and morning, although a moderate effect size was found for all parameters (except iEMG) with higher values observed in the evening (Table 1).

**Table 1:** Mean and SD for performance time, power output (PO), aerobic power (Paer), anaerobic power (Pan), integrated electromyography (iEMG), oxygen uptake (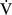 O_2_) and heart rate (HR) during a 1000-m cycling TT performed in the morning or in the evening (n = 9).

Variables	MORNING	EVENING	Effect size (*η*_p_^2^)
Time (s)	94.7±10.9	88.2±8.7*	0.63
PO (W)	349.2±93.9	388.5±104.4	0.37
Paer (W)	162.2±24.4	167.3±30.6	0.48
Pan (W)	187.0±92.9	221.2±95.2	0.34
iEMG (%MVC)	83.8±27.2	84.0±34.0	0.01
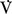 O_2_ (L.min^−1^)	2.91±0.25	2.97±0.37	0.34
HR (bpm)	146±29	154±14	0.32

* Significantly lower than morning (p<0.05).

**Figure 2:**
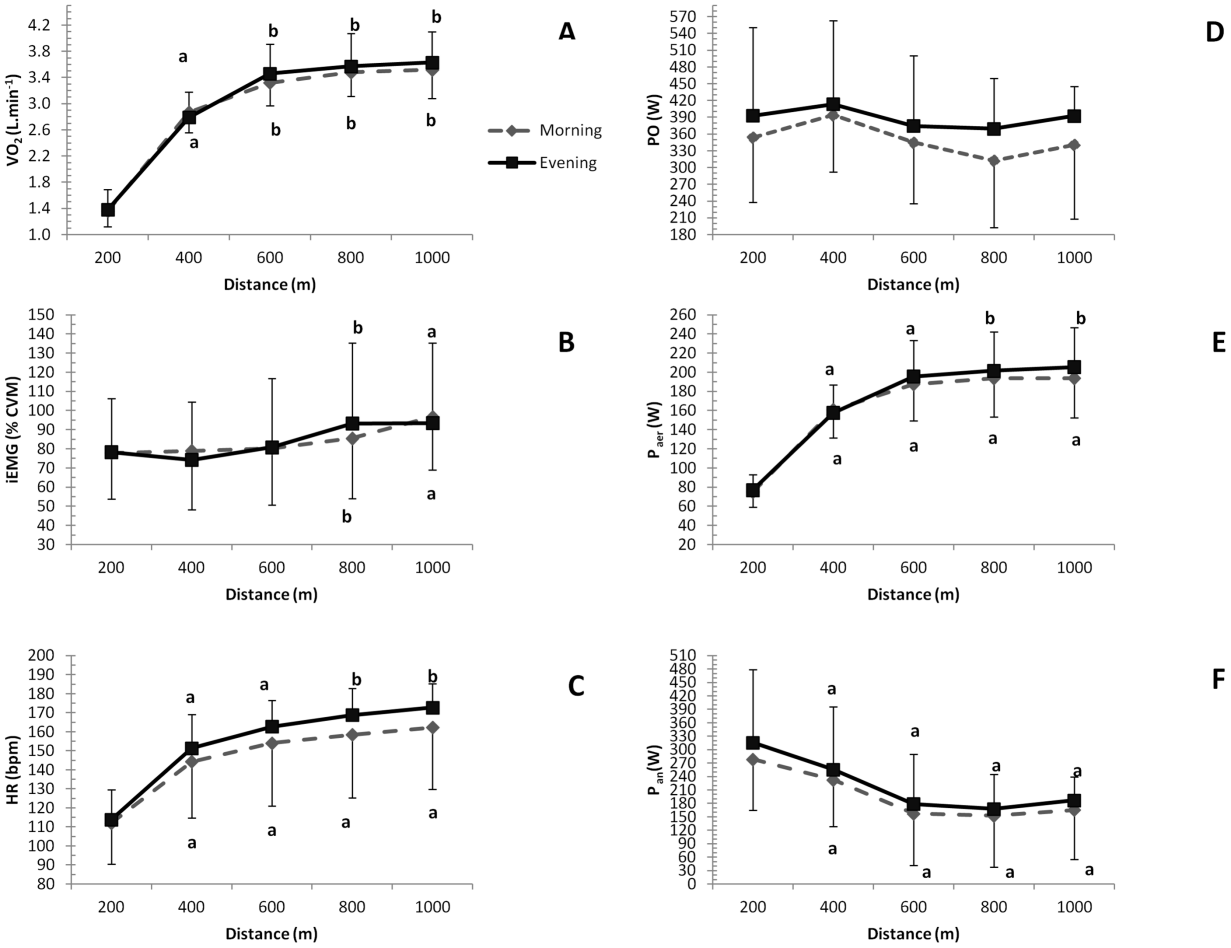
Mean and SD for oxygen uptake (A), integrated electromyography (B), heart rate (C), power output (D), aerobic power contribution (E), anaerobic power contribution (F) for each 200-m interval during the 1000-m cycling TT performed in the morning or in the evening. ^a^ Significantly different than the 200-m value for the same time of day; ^b^ Significantly different than the 400-m value for the same time of day; 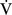 O_2_ and HR increased exponentially with distance for times of day (p<0.05), but there was a tendency for Paer values at 600 and 800 m, and 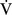 O_2_ at 600 and 1000 m, to be higher in the evening than in the morning (p<0.10).

The PO, Paer, Pan, iEMG, 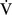 O_2_ and HR response to the 1000-m cycling TT in the morning and the evening are displayed in figure 2. There was no main effect for time of day or interaction for all of these variables (all p>0.10), but there was a tendency for Paer and 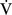 O_2_ values at the 600- and 800-m sections to be higher in the evening than in the morning (p = 0.07 and 0.06, respectively). There was a main effect of distance for Paer, Pan, iEMG, 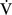 O_2_, and HR (all p<0.01), but not for PO (p>0.10).

### Hormones, glucose and lactate

The hormone, glucose and lactate response to the 1000-m cycling TT in the morning and evening are displayed in Table 2. There was no significant main effect for time of day or interaction for glucagon, norepinephrine or epinephrine (all p>0.10). There was a significant main effect of time for these hormones (all p<0.001), with values immediately after the TT being higher than baseline and 60′ post-TT for both trials, but without a significant difference between the 60′ post-TT and baseline (p>0.10).

**Table 2:** Mean and SD for hormones, glucose and lactate concentrations at baseline, immediately (Post-TT), and sixty minutes after (60′ Post-TT) a 1000-m cycling TT performed in the morning or in the evening (n = 9).

	Baseline		Post-TT		60′ Post-TT	
Hormones	Morning	Evening	Morning	Evening	Morning	Evening
Glucagon (pg.mL^−1^)	2.45±0.71	2.62±0.69	11.38±5.20^a^	9.39±4.15^a^	3.25±1.49^b^	3.65±1.32^b^
Insulin (IU.mL^−1^) (Log)*	1.59±0.96	1.31±0.71	1.74±1.21	1.10±1.29	1.38±0.61	1.20±0.94
GH (ng.mL^−1^) (Log)**	0.65±0.71	0.84±0.90	1.32±0.91^a^	1.77±1.17^a^	0.85±0.63^Ω^	1.68±1.03
Cortisol (ug.dL^−1^) (Log)*	2.49±0.33	2.07±0.87	2.62±0.43^a,Ω^	2.26±0.81	2.52±0.52^Ω^	2.05±0.68^b^
Total TE (ng.dL^−1^) (Log)*	6.59±0.89	6.41±0.26	6.21±0.26	6.10±0.18	6.25±0.21^b,Ω^	5.92±0.27^b^
Free TE (ng.dL^−1^) (Log)*	2.16±0.23	1.92±0.18	2.23±0.26^a^	2.03±0.12^a^	2.10±0.20^b,Ω^	1.74±0.17^bΩ^
Norepinephrine (pg.mL^−1^) (Log)	5.23±0.47	5.58±0.39	6.93±0.56^a^	6.66±0.66^a^	5.74±0.51^b^	5.84±0.67^b^
Epinephrine (pg.mL^−1^) (Log)	4.04±0.59	4.29±0.81	4.98±0.63^a^	4.89±0.50^a^	4.36±0.51^b^	4.31±0.73^b^
Glucose (mmol.L^−1^) (Log)**	1.50±0.22	1.71±0.13	1.63±0.17	1.73±0.16	1.50±0.13	1.68±0.23
Lactate (mmol.L^−1^)	2.45±0.71	2.62±0.69	11.38±5.20^a^	9.39±4.16^a^	3.25±1.49^b^	3.65±1.32^b^

*,** Main effect for time of day (*Morning significantly higher than evening; ** Morning significantly lower than evening). ^a^ Significantly higher than baseline. ^b^ Significantly lower than post-TT. ^Ω^ Significantly different than evening at the same time point. GH: growth hormone; TE: testosterone.

Insulin, cortisol, and total and free testosterone were higher in the morning than in the evening (p = 0.05, 0.05, 0.03 and 0.01, respectively), while GH was higher in the evening than in the morning (p = 0.02). In addition, the GH values 60′ post-TT were significantly lower in the morning than in the evening (p = 0.023), while total testosterone values at the same time point were higher in the morning than in the evening (p = 0.005). Baseline GH values were lower than immediately post-TT (p<0.05) for both morning and evening trials, but without a significant difference between post-TT and 60′ post-TT (p>0.10). Total testosterone immediately after the TT was higher than the 60′ post-TT (p<0.05) in both trials, but without a significant difference between post-TT and baseline or between 60′ post-TT and baseline (p>0.10). Similar results were obtained for free testosterone, except that values post-TT were also higher than baseline for both trials (Table 2).

The plasma glucose was lower in the morning than in the evening (p = 0.04). However, there was no significant effect of time or an interaction effect (p>0.10). The plasma lactate increased with the exercise but returned to baseline values after 60 min of recovery for both conditions (no time of day or interaction effect, all p>0.10).

**Figure 3:**
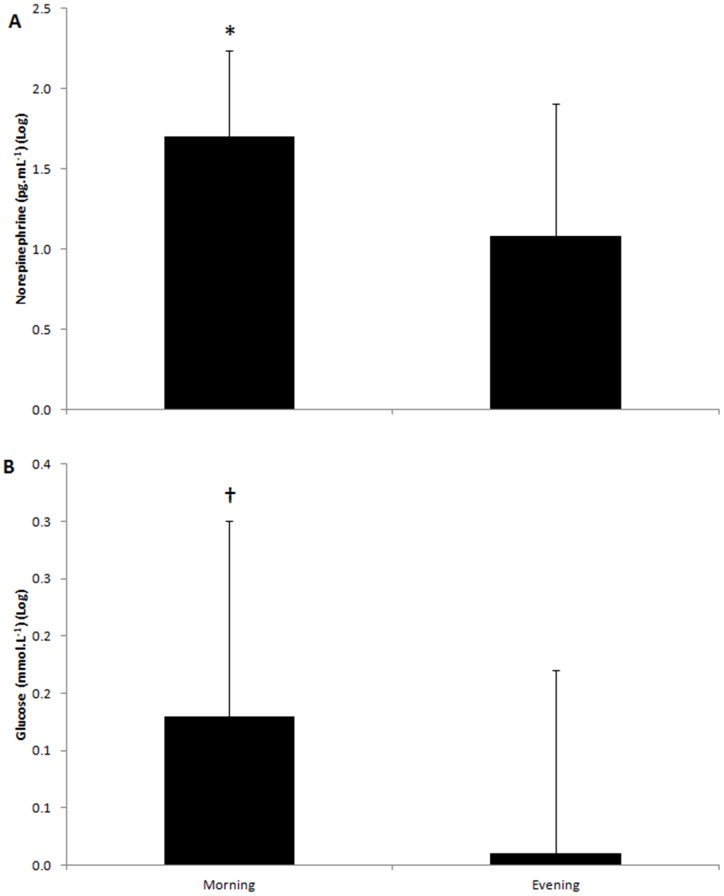
Mean and SD for norepinephrine (A) and glucose (B) response to the exercise (post-TT less baseline). * Significantly higher in the morning than in the evening. † Tendency to be higher in the morning than in the evening (p<0.10).

The normalized norepinephrine response to the exercise (post-TT less baseline) was significantly higher in the morning than in the evening (Figure 3A). The normalized glucose response to the exercise tended also to be higher in the morning than in the evening (Figure 3B). There was no difference between the morning and evening response, or the recovery from the exercise, for any other variable.

### Test order effect

There was no test order effects for any of the variables investigated (all p>0.10).

## Discussion

The main purpose of this study was to determine the effect of time of day on performance, pacing, energy system contribution, and metabolic and hormonal responses during a 1000-m cycling TT. The main findings of the present study were: 1) a better performance (shorter TT times) in the evening than in the morning, which was not accompanied by a significant alteration in the aerobic or anaerobic contribution; 2) Insulin, cortisol, and free and total testosterone were lower in the evening, while GH and plasma glucose concentration were higher in the evening. However, the norepinephrine and glucose responses to the exercise were higher in the morning.

In the present study, the time for amateur, recreational cyclists to complete the 1000-m cycling TT was ∼6.5 s (∼6.9%) quicker in the evening than in the morning. This represented a large effect size (*η*_p_^2^ = 0.63), suggesting that time of day can affect performance during a short-distance TT. This improved performance in the evening is in agreement with previous reports of better performance in the evening during a 30-s Wingate test [5], a 60-s all-out test [14], a repeated-sprint test [9], and a high-intensity (95% VO_2_max), constant-load exercise [6]. We had hypothesized that this improvement on performance in the evening might be associated with parallel changes in pacing and energy system distribution. Although visually the power output appears to be maintained at a higher value over the last 400 m in the evening when compared to morning (figure 2), these alterations failed to reach statistical significance. In addition, no apparent differences between the morning and evening Pan and Paer profiles during the trial were identified. Instead, there were moderate effect sizes for the mean Pan and Paer, with higher values observed in the evening (table 1). These increased mean Pan and Paer in the evening accompanied a moderate effect size for mean PO and a large effect size to time performance in favor of evening (table 1). Together, these results suggest that improvement in performance in the evening trial was not caused either by alteration in a unique energy system (i.e. aerobic or anaerobic), or by a rise in the aerobic or anaerobic energy supply at a given time point of the trial. Instead, performance in the evening seemed to be improved due a concomitant and maintained increase in aerobic and anaerobic contributions throughout the trial. These results are in agreement with other studies showing that an enhanced performance in the evening is accompanied by a slightly increase in both the aerobic and anaerobic contribution [4], [7].

In parallel with a different performance between the morning and evening, we also observed hormonal and metabolic differences between the times of day. Insulin was increased and GH was reduced in the morning. Plasma glucose was also reduced in the morning. On the other hand, cortisol levels were increased in the morning. Cortisol levels peak in the morning and decrease linearly throughout the day with the lowest value being reached at ∼8:30 pm [25]. This elevated cortisol in the morning is indicative of an increased physiological stress, and it would be expected to elevate plasma glucose [26]. However, as insulin reduces plasma glucose levels [27], any effect of cortisol on plasma glucose may have been off-set. In addition, the circadian profile of GH produces a peak concentration in the night during the first two hours of sleep, but is reduced in the morning [28]. A reduced GH concentration in the morning may have contributed to the reduced plasma glucose concentration [28], [29], [30]. We hypothesise therefore that elevated insulin, as well as reduced GH concentrations in the morning, may not create an optimal metabolic *milieu* to meet the best performance It is also interesting to note that the norepinephrine response to exercise was amplified in the morning compared to the evening, and this was accompanied by a tendency for a greater plasma glucose response to the exercise. This higher norepinephrine response to the exercise in the morning may have occurred to counterbalance the reduced plasma glucose concentration. Norepinephrine can increase plasma glucose by either its direct effect on hepatic glucogenolysis [31], [32] or indirectly by increased mobilization of free fatty acids [33].

We also found that free and total testosterone levels were higher in the morning than in the evening, and both increased with exercise at both times of day. It has been documented that testosterone presents a biorhythmic effect with the highest values in the morning (08:00 am) and the lowest in the evening (08:00 pm), but that the acute response to exercise is similar at different times of the day [12], [34]. Little attention has been given to the metabolic effects of testosterone and its relation with fatigue. Positive correlations between the basal level of testosterone and sprinting and explosive power performances have been found [35]. Elevated testosterone concentration may be involved with an increased neuromuscular efficiency [35] and enhanced Ca^2+^ handling mechanism in the fast-twitch muscle fibers [15]. Therefore, while an elevated testosterone in the morning could partially compensate the negative metabolic *milieu*, perhaps via signaling process into the cells, further studies investigating the acute molecular effect of testosterone and it relationship with biorhythms are necessaries to confirm this hypothesis.

It is important to acknowledge that we recruited recreational cyclists and these results should not be extrapolated to other groups such as highly-trained athletes or sedentary/clinical populations. Further studies should be performed investigating if the effects found in the present study are reproducible in these populations. It is also important to underline that competitions do not always occur in the evening. However, training can be performed in both the morning and evening. It is necessary to recognize that training may have to be performed under unfavorable conditions, but as close as possible to the competition environment and conditions (e.g. if the competition is in the morning). Corroborating with this last statement, Edwards et al. [36] observed that performance was better when the previous training was performed in the early morning (07:00 AM) than in the noon (12:00 AM). These results suggest that exercise performance may be acutely altered by the habitual timing of training, and underline the necessity to understand the effects of exercise and training at different time of days.

In conclusion, performance was impaired in the morning compared with the evening, but it was not associated with a clear alteration in pacing, an aerobic and anaerobic energy supply distribuition. Morning exercise was performed in a less favorable metabolic *milieu* (i.e. elevated insulin and cortisol, and reduced plasma glucose levels), combined with an exacerbated norepinephrine and plasma glucose response to the exercise.
